# It takes two—or more—to tango: Revisiting the role of dopamine transporter oligomerization

**DOI:** 10.1016/j.jbc.2021.100629

**Published:** 2021-04-27

**Authors:** Ulrik Gether, Harald H. Sitte

**Affiliations:** 1 Department of Neuroscience, Faculty of Health and Medical Sciences, Maersk Tower 7.5, University of Copenhagen, Copenhagen, Denmark; 2 Center for Physiology and Pharmacology, Institute of Pharmacology, Medical University Vienna, Vienna, Austria

**Keywords:** dopamine transporter, monoamine transporters, dimerization, oligomerization, allosteric modulation, endocytosis, AL, AIM-100-like compound, DAT, dopamine transporter, NET, norepinephrine, NSS, neurotransmitter:sodium symporter, SERT, serotonin, TM, transmembrane

## Abstract

The dopamine transporter utilizes the transmembrane sodium gradient to mediate reuptake of dopamine from the extracellular space. The dopamine transporter can form dimers and possibly also higher order structures in the plasma membrane, and this oligomerization has been implicated in both trafficking and transport. However, we still do not fully understand its biological importance. A study by Sorkina *et al.* now describes a series of small molecules that link transporter conformation to oligomerization and endocytosis, providing an interesting step forward in an intricate dance.

The dopamine transporter (DAT) belongs to the large class of neurotransmitter:sodium symporters (NSS) (also referred to as solute carrier 6 transporter family), together with other neurotransmitter transporters, such as the norepinephrine (NET), serotonin (SERT), γ-aminobutyric acid, and glycine transporters (1, 2). These transporters play an essential role in clearing released transmitter from the extracellular space and thereby for regulating neurotransmitter homeostasis using the Na^+^-gradient to translocate their respective substrates across the plasma membrane (1, 2). NSS proteins have received substantial attention as prime targets for several medicines (*e.g.*, attention deficit hyperactivity disorder medication, antidepressants, antiepileptics) and for drugs of abuse (*e.g.*, cocaine, amphetamines, cocaine, and MDMA [ecstasy]) (1, 2).

“It takes two to tango” is a classical cliché that was already suggested many years ago to describe the putative importance of dimerization of DAT and the two other monoamine transporters, SERT and NET (3). Now, there is almost overwhelming evidence that DAT teams up into dimers or oligomers to exert its function in the plasma membrane of the neurons in which it is expressed (for review see (4)). Recently, single molecule fluorescence microscopy has enabled the most direct assessment of DAT and SERT oligomer formation in the plasma membrane yet. These data showed that DAT and SERT oligomers were highly stable over time, but a surprising difference in oligomerization stoichiometry was seen between the two transport proteins with a mixture of monomers and oligomers (up to pentamers and higher order oligomers) observed for SERT (5) and a mix of monomers and dimers observed for DAT (6). Such a difference between highly homologous transporters is puzzling and might suggest different functional implications of oligomer formation for the different transporters. Indeed, oligomerization has been suggested to serve several different roles in NSS proteins' function. These encompass facilitation of cellular trafficking of the transporters, including both trafficking to and from the plasma membrane, as well as functional cooperativity between protomers and, for the monoamine transporters, promotion of reverse transport by substrates such as amphetamine and its congeners (for review see (3)). Of particular interest in relation to the study by Sorkina *et al.* (7), it has also been shown that ligands targeting DAT, like amphetamines, can affect oligomerization. The data are somewhat blurry as *in vivo* studies suggested that repeated administration of metamphetamine to mice increases DAT oligomerization (8), whereas *in vitro* studies rather have supported that amphetamines disperse oligomer formation (9). Nevertheless, the data underscore that oligomerization might not only be important for the pharmacological action of ligands such as amphetamine but also ligands by themselves can have the propensity to affect oligomerization of this class of proteins.

In two consecutive studies, Sorkina *et al.* explore this concept by describing new ligands that promote DAT oligomerization and affect DAT function and trafficking (7, 10). In their previous study, the authors discovered that furopyrimidine AIM-100, an inhibitor of the activated CDC42 tyrosine kinase (ACK1/TNK2), dramatically increased DAT oligomerization and clustering in the membrane and endocytosis (10). The authors suspected that these observations were independent of ACK1 activity, but because ACK1 has been proposed to regulate DAT endocytosis, they could not be sure without more specific compounds. Moreover, the mechanism by which AIM-100 caused these outcomes was unclear. To learn more, Sorkina *et al.* (7) now screen a small library of AIM-100 analogs searching for AIM-100-like compounds (ALs) with the same effect on DAT but no effect on ACK1. Importantly, they successfully identify several compounds with these properties. The compounds display a remarkable ability to promote formation of SDS-resistant oligomers of DAT that appear to be dominated by a DAT trimer (MW∼200 kD). The formation of oligomers in response to ALs is supported by Förster resonance energy-transfer measurements, and it is demonstrated both by live fluorescence microscopy and surface biotinylation experiments that the compounds induce robust DAT endocytosis (7). Through mutational analysis aimed at delineating the mechanism underlying the effects of ALs, Sorkina *et al.* (7) generate an interesting quadruple mutant where four residues from transmembrane (TM) segments 4 and 9 are replaced with their SERT counterparts (the “TM4-9” mutant). This mutant shows a striking reduction in ALs-induced oligomerization and endocytosis. In addition, computational simulations indicate that the mutations promote a more outward-facing conformation of the transporter, which is further supported by an apparent increase in binding affinity for cocaine and dopamine by the mutant. In contrast, truncation of the N terminus, which should bias the transporter toward the inward-facing conformation and hamper ligand uptake, is found to enhance the ability of the ALs to induce oligomer formation and endocytosis. Based on these data, together with additional computational simulations, the authors propose a model in which transporter endocytosis and oligomer formation are favored by an inward-facing conformation of the transporter (Fig. 1). The ALs are thus proposed to facilitate oligomerization and endocytosis *via* their ability to stabilize this conformation by binding to the trimeric interface, which is disrupted in the TM4-9 mutant (7).

**Figure 1:**
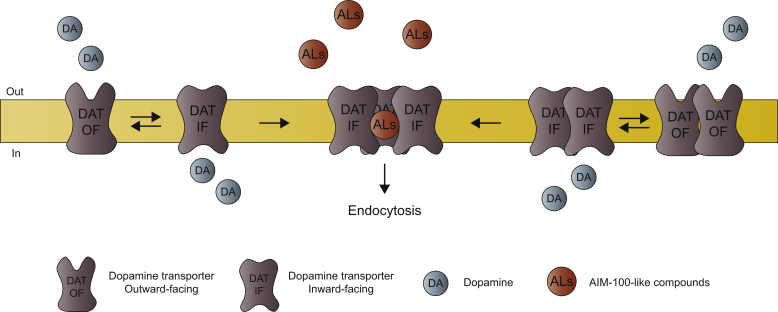
**DAT is thought to exist in the plasma membrane primarily either as a monomer or as a dimer.** The monomers and dimers appear stable over time with little exchange (6). To transport dopamine (DA), the transporter switches back and forth between an outward-facing conformation (OF), capable of binding DA from the extracellular side, and an inward-facing conformation (IF) that allows release of DA to the intracellular environment. The AIM-100-like compounds (ALs) identified by Sorkina *et al.* (7, 10) show a remarkable ability to promote formation of DAT trimers by binding at a suggested trimeric interface between the transporter protomers. The binding is favored by the inward-facing conformation of DAT and strongly promotes DAT oligomerization, clustering, and endocytosis. In this way, the ALs affect DAT function in a way different from any other known ligand targeting DAT. DAT, dopamine transporter.

These intriguing ideas also reveal some unanswered questions. Is it possible, for example, that the effect of the ALs is unrelated to the “real” importance of DAT oligomerization? It may be difficult to exclude that the compounds somehow promote formation of unnatural, SDS-resistant higher order structures (trimers) that never would exist under normal circumstances. As mentioned above, the application of single-molecule approaches to DAT did not reveal any trimers but only dimers and monomers in the plasma membrane of DAT expressing cells (6). However, as noted by Sorkina *et al.*, the immobile trimers may not have been detected by the single-molecule approach, suggesting further research is needed. The proposed binding site at the trimeric interface might also be considered speculative in the absence of direct structural insights, and it cannot be excluded that the ALs have yet unknown effects as they were only tested for activity against ACK1. Nevertheless, it seems clear that the study by Sorkina *et al.* identifies a number of ligands that *via* an allosteric mechanism appear to promote trimer formation and thereby affect DAT function in a way different from any other known ligand targeting DAT or any others NSS protein (7). This could open for entirely new ways of pharmacologically manipulating DAT and perhaps design new therapeutics that allosterically modulate DAT activity. Thus, it might be true that sometimes and—at least for the action of ALs—it takes more than two to tango.

## Conflict of interest

The authors declare no conflicts of interest with the contents of this article.
